# Rapid and long-lasting antidepressant-like effects of ketamine and their relationship with the expression of brain enzymes, BDNF, and astrocytes

**DOI:** 10.1590/1414-431X202010107

**Published:** 2020-12-18

**Authors:** G.S.B. Viana, E.M. do Vale, A.R.A. de Araujo, N.C. Coelho, S.M. Andrade, R.O. da Costa, P.E.A. de Aquino, C.N.S. de Sousa, I.S. de Medeiros, S.M.M. de Vasconcelos, K.R.T. Neves

**Affiliations:** 1 Departamento de Fisiologia e Farmacologia, Faculdade de Medicina, Universidade Federal do Ceará, Fortaleza, CE, Brasil; 2 Departamento de Biofisiologia, Faculdade de Medicina Estácio de Juazeiro do Norte, Juazeiro do Norte, CE, Brasil

**Keywords:** Ketamine, Antidepressant-like effects, Forced swimming test, Neuroplasticity

## Abstract

Ketamine (KET) is an N-methyl-D-aspartate (NMDA) antagonist with rapid and long-lasting antidepressant effects, but how the drug shows its sustained effects is still a matter of controversy. The objectives were to evaluate the mechanisms for KET rapid (30 min) and long-lasting (15 and 30 days after) antidepressant effects in mice. A single dose of KET (2, 5, or 10 mg/kg, *po*) was administered to male Swiss mice and the forced swim test (FST) was performed 30 min, 15, or 30 days later. Imipramine (IMI, 30 mg/kg, *ip*), a tricyclic antidepressant drug, was used as reference. The mice were euthanized, separated into two time-point groups (D1, first day after KET injection; D30, 30 days later), and brain sections were processed for glycogen synthase kinase-3 (GSK-3), histone deacetylase (HDAC), brain-derived neurotrophic factor (BDNF), and glial fibrillary acidic protein (GFAP) immunohistochemical assays. KET (5 and 10 mg/kg) presented rapid and long-lasting antidepressant-like effects. As expected, the immunoreactivities for brain GSK-3 and HDAC decreased compared to control groups in all areas (striatum, DG, CA1, CA3, and mainly pre-frontal cortex, PFC) after KET injection. Increases in BDNF immunostaining were demonstrated in the PFC, DG, CA1, and CA3 areas at D1 and D30 time-points. GFAP immunoreactivity was also increased in the PFC and striatum at both time-points. In conclusion, KET changed brain BDNF and GFAP expressions 30 days after a single administration. Although neuroplasticity could be involved in the observed effects of KET, more studies are needed to explain the mechanisms for the drug’s sustained antidepressant-like effects.

## Introduction

Major depressive disorder (MDD) is a severe and debilitating psychiatric disease, for which the available antidepressant drugs do not always show a good response in a large percentage of the depressive population (1). In contrast to existing antidepressants, the fast-acting effect of ketamine (KET) provides relief for patients with MDD, including those at risk of suicide (2).

Although the inhibition of glycogen synthase kinase-3 (GSK-3 beta) modulates m-TOR signaling and may potentially augment the effects of antidepressants, such as KET, it is unclear whether GSK-3 directly mediates this drug effect. GSK-3 is linked to some neuropsychiatric disorders, including depression, where this enzyme is dysregulated (3). Neurotransmitter systems, as the serotonergic, dopaminergic, cholinergic, and glutamatergic ones, also regulate GSK-3 activity (4). In addition, GSK-3 is shown to be an important target for KET rapid antidepressant effects, mediated by enhanced α-amino-3-hydroxy-5-methyl-4-isoxazolepropionic acid (AMPA), resulting from the drug NMDA inhibition (5). Several classes of serotonin-modulating drugs, such as antidepressants, regulate GSK-3 by inhibiting its brain activity. This inhibition reinforces the importance of GSK-3 as a potential therapeutic target in neuropsychiatric diseases associated with an abnormal serotonin function (6).

A neural mechanism involved in the antidepressant effects of KET is the AMPA receptor stimulation, which has been shown to mediate an increase in the brain extracellular levels of serotonin, as also shown by our group (7). However, mechanisms other than NMDA receptor inhibition play a key role in the antidepressant effect of KET, suggesting that the KET antidepressant effect goes beyond the NMDA receptor inhibition and AMPA receptor activation. Recent data (8) demonstrate that GluN2B-NMDA receptors on GABA interneurons are the initial cellular trigger for the rapid antidepressant actions of KET.

Another enzyme potentially important as a therapeutic target for antidepressant drugs is histone deacetylase (HDAC). Preclinical studies have demonstrated changes in brain gene expression in animal models of depression, and some of these models point to the antidepressant efficacy of HDAC inhibitors. For instance, Lu et al. (9), showed that HDAC inhibitors decrease the expression of glial fibrillary acidic protein (GFAP) within damaged tissue, following traumatic brain injury.

Chronic administration of antidepressant drugs is known to increase neurotrophins, including brain-derived neurotrophic factor (BDNF) (10). This leads to enhanced neuronal plasticity, such as neurogenesis, synaptogenesis, and neuronal maturation. Thus, BDNF may be considered a useful marker for clinical response or improvement of depressive symptoms. The activation of the mammalian target of the rapamycin (mTOR) pathway by KET enhances translation of BDNF in the hippocampus (11,12). Astrocytes were demonstrated to be the main recipient of neuronal-expressed BDNF, which, when taken up by astrocytes, mediates physiological effects on these cells. Furthermore, astrocyte dysfunction can lead to alterations in neuronal functions and to several brain disorders, including depression (13).

Evidence (14) indicates that direct AMPA receptor activation may play an important role in both the rapid and sustained antidepressant-like effects of ketamine in animal models of depression, although other mechanisms might be involved in the sustained action. We showed that KET exhibits antinociceptive and anti-inflammatory effects (15) and, in addition, we also observed that the monoaminergic pathways and inhibition of monoamine transporters are involved with the antidepressant-like effect of KET. Furthermore, our present results indicated the close involvement of GSK-3 and HDAC inhibitions and the blockade of inflammatory processes with the drug’s antidepressant-like action.

Thus, the objectives of this study were to determine the acute and sustained KET antidepressant effects, evaluated by the forced swimming test (FST), focusing on KET relationships with brain enzymes, such as GSK-3 and HDAC. Considering the importance of BDNF and astrocytes for depression and neural plasticity, we also aimed at studying BDNF and GFAP, at two time-points (day 1, D1; 30 days later, D30) after a single KET injection, in order to have some insight on the rapid and, especially, the long-lasting effects of KET.

## Material and Methods

### Drugs and reagents

Ketamine (racemic form) was from König (Brazil). Antibodies for immunohistochemistry assays were from Sigma-Aldrich (USA) or Abcam (UK). All other reagents were of analytical grade.

### Animals

Male Swiss mice (30 g) from the Animal House of the Faculty of Medicine Estácio de Juazeiro do Norte (Estácio/FMJ) were maintained at 24±2°C, in a 12-h dark/light cycle, with standard food and water *ad libitum*. The study was approved (#2014-004) by the Estácio/FMJ Ethics Committee for Animal Experimentation. All experiments followed the ethical principles established in the Guide for the Care and Use of Laboratory Animals, USA (2011).

### Forced swim test (FST)

**Figure 1:**
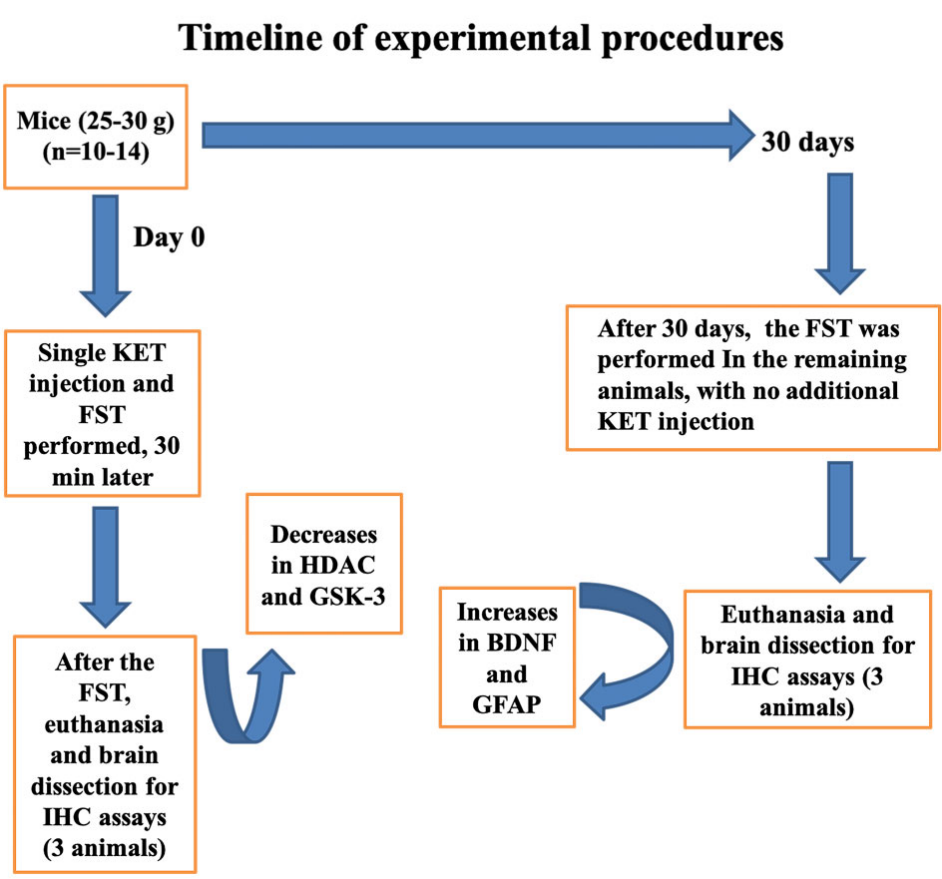
Study timeline. KET: ketamine; FST: forced swim test; IHC: immunohistochemistry; BDNF: brain derived neurotrophic factor; GFAP: glial fibrillary acidic protein;GSK-3: glycogen synthase kinase; HDAC: histone deacetylase.

This rodent behavioral test is used for the evaluation of antidepressant drugs and the antidepressant efficacy of new compounds. The test is based on the observation that, when the animals are subjected to a stressful situation with no possibility for escaping, they adopt a posture of immobility after an initial period of agitation. The reduction of this immobility time is suggestive of an antidepressant action and is the parameter used for the antidepressant-like effect (16). A glass cylinder (30 cm height × 20 cm diameter) is filled with water (15 cm from the bottom, at 25°C). Ketamine (KET: 2, 5, or 10 mg/kg, *po*) or imipramine (IMI: 30 mg/kg, *ip*) as the test reference drug was administered to mice. The control group was administered 0.1 mL/100 g distilled water, *po*. Thirty minutes after KET injections, each mouse was placed individually into the cylinder. After 2 min, the immobilization time was recorded for 5 min. In order to demonstrate the long-lasting effect of KET, a single dose of the drug was also injected and the FST performed 15 or 30 days later. IMI was always administered 1 h before testing. At the end, the apparatus and testing area were cleaned with 70% ethanol before starting the next test. Figure 1 shows the timeline of experimental procedures, including the FST and immunohistochemical assays (performed at two time-points, 1 day and 30 days, after the FST, with no additional KET injection). The animals remained untreated in this 30-day period.

### Immunohistochemical (IHC) assays for brain GSK-3, HDAC, BDNF, and GFAP

Sections (5-µm thickness) from brain areas (hippocampus, striatum, prefrontal cortex (PFC), and temporal cortex) of three animals per group were fixed in 10% buffered formaldehyde for 24 h followed by a 70% ethanol solution. The brain sections were obtained (stainless steel brain matrices, 1.0 mm) after 60 min, in the two time-point protocols, named D1 and D30, following the administration of a single intraperitoneal dose of KET and after performing the FST. Then, the sections were embedded in paraffin wax for processing on appropriate glass slides. These were placed in the oven at 58°C for 10 min followed by deparaffinization in xylol, rehydration in alcohol at decreasing concentrations, and washing in distilled water and PBS (0.1 M sodium phosphate buffer, pH 7.2) for 10 min. The endogenous peroxidase was blocked with a 3% hydrogen peroxide solution, followed by incubation with the appropriate primary anti-antibody for GSK-3 (ab75745, rabbit polyclonal antibody), HDAC1 (ab53091, rabbit polyclonal antibody), both from Abcam, BDNF (rabbit polyclonal antibody), and GFAP (goat polyclonal antibody), both from Sigma-Aldrich. After 2 h at room temperature in a moist chamber, the slides were washed with PBS (3 times, 5 min each) and incubated with the biotinylated secondary antibody for 1 h at room temperature. Then, they were washed again with PBS and incubated with streptavidin-peroxidase for 30 min at room temperature. After another wash in PBS, they were incubated in a 0.1% DAB solution (in 3% hydrogen peroxide). Finally, the slides were washed in distilled water, dehydrated in alcohol (at increasing concentrations), diaphanized in xylol and mounted on Entelan^®^ for optic microscopy examination. The data (from 3 animals per group) were quantified with the ImageJ software (National Institutes of Health, USA).

### Statistical analysis

The results are reported as means±SE. For statistical analyses, one-way ANOVA followed by Tukey as the *post-hoc* test for multiple comparisons were used (GraphPad Prism, version 7.0, USA). The immunohistochemical data (absorbance) were calculated with the ImageJ software (NIH). Differences were considered significant at P<0.05.

## Results

### Forced swim test

**Figure 2:**
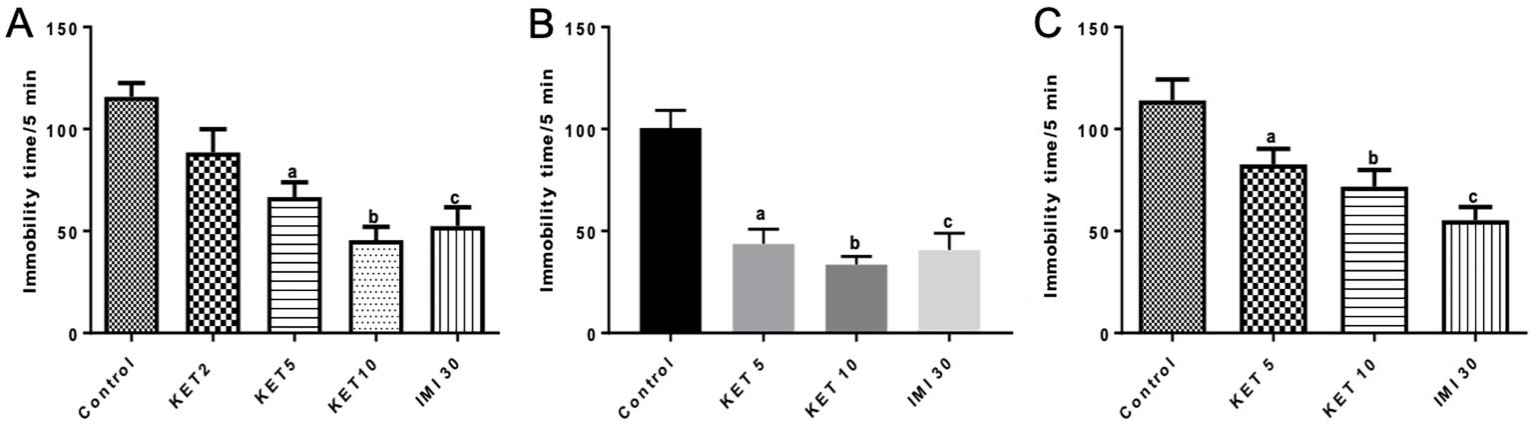
Antidepressant-like effects of single administrations of ketamine (KET, 5 and 10 mg/kg) evaluated by the forced swim test in male mice (7 to 10 animals per group) 30 min (**A**), 15 days (**B**), and 30 days (**C**) later. Data are reported as means±SE. **A**, ^a,b,c^P<0.05 *vs* Control; **B** and **C**, ^a,b,c^P<0.001 *vs* Control (one-way ANOVA and Tukey as the *post hoc* test).

We showed dose-dependent decreases of 34, 46, and 63% in the immobility time (s) for 5 min, after 30 min (7 to 12 animals per group) of a single KET administration, at the doses of 2, 5, and 10 mg/kg, respectively, in relation to the control group. Imipramine showed a 58% decrease of the immobility time. The F-statistic and its associated degrees of freedom and P-value are F(4,42)=14.53, P<0.0001 (Figure 2A). Similar results were observed at 15 days (9-13 animals per group) after a single administration of KET (55 and 62% decreases, for the doses of 5 and 10 mg/kg, respectively). IMI (n=7) decreased the immobility time by 53% in relation to controls (Figure 2B). However, measurements of the immobility time, performed at D30 (13 to 16 animals per group) after a single KET injection showed no clear dose-dependent relationship, with lower decreases (31 and 40%) after the doses of 5 and 10 mg/kg, respectively. IMI-30 (n=6 animals) decreased the immobility time by 54% [F(3,44)=9.042, P<0.0001] (Figure 2C). Similar results were observed 30 days after a single KET administration, as evaluated by the tail suspension test (data not shown).

### Immunohistochemical results for GSK-3

**Figure 3:**
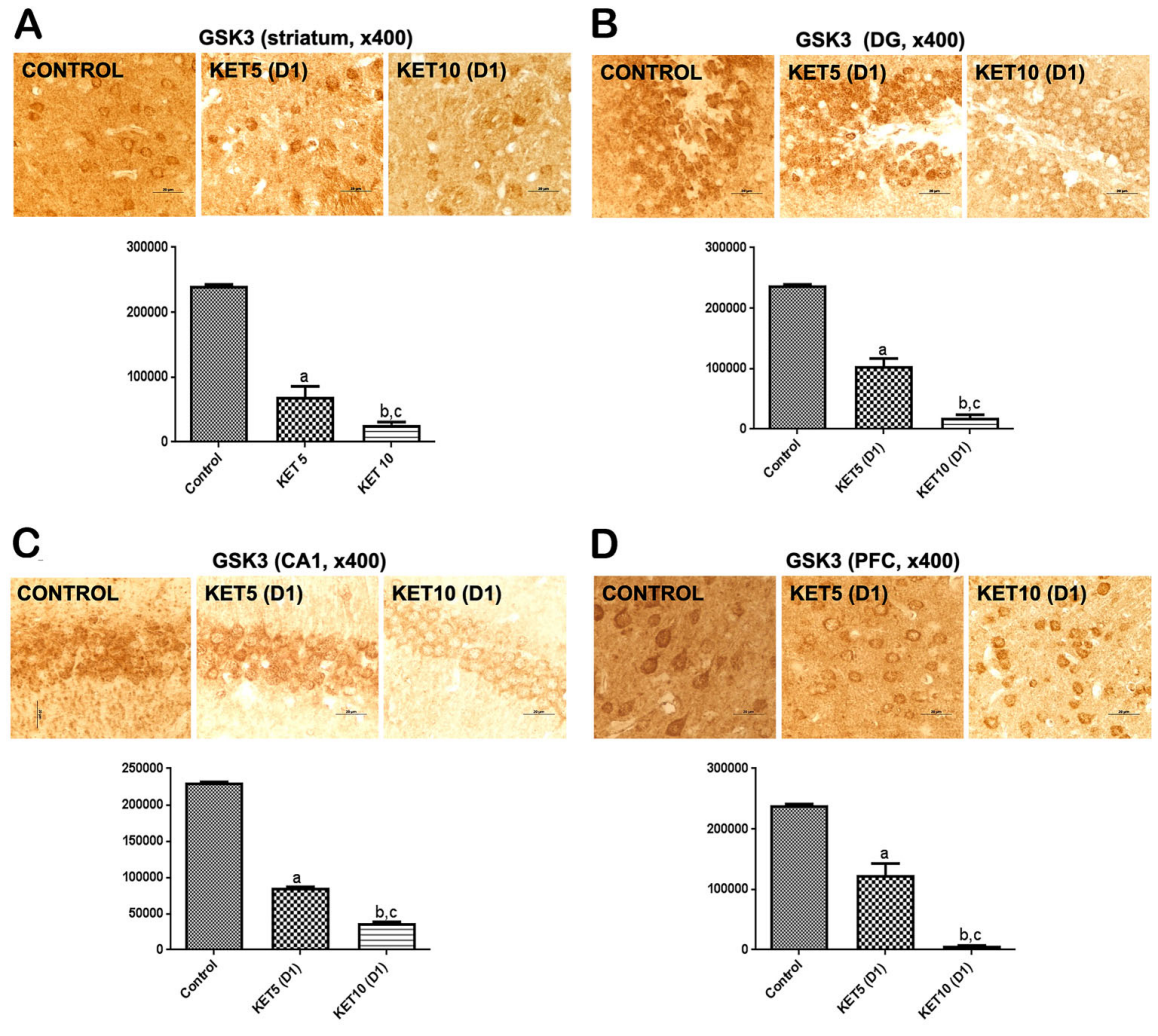
Single administrations of ketamine (KET, 5 and 10 mg/kg, *ip*) significantly reduced glycogen synthase kinase (GSK-3) immunoreactivities in **A**) striatum, **B**) dentate gyrus (DG), **C**) CA1 hippocampal subfield, and **D**) prefrontal cortex (PFC), in a dose-dependent manner compared with controls (3 animals per group). Representative photomicrographs, at ×400 magnification (scale bar: 20 μm), taken at day 1 (D1) protocol. The graphs show the relative absorbance determined by ImageJ software (NIH, USA). Data are reported as means±SE. ^a,b^P<0.01 *vs* Control; ^c^P<0.01 *vs* KET 5 (one-way ANOVA and Tukey as the *post hoc* test).

Greater reductions in GSK-3 immunoreactivities were demonstrated after acute intraperitoneal KET injection in all brain areas studied. Thus, 64 and 92% decreases were observed in the striata 60 min after the doses of 5 and 10 mg/kg, respectively, compared with the control group [(F(2,9)=181.0, P<0.0001] (Figure 3A). Also, in the dentate gyrus (DG), the decreases were 56 and 92%, respectively, [F(2,9)=176.9, P<0.0001] (Figure 3B). The decreases in the hippocampus CA1 area were 63 and 84%, respectively, for the same doses [F(2,6)=728.6, P<0.0001] (Figure 3C). Interestingly, even greater decreases (73 and 99%) were seen in the PFC after the acute KET administrations of 5 and 10 mg/kg, respectively [F(2,9)=186.1, P<0.0001] (Figure 3D).

### Immunohistochemical results for brain HDAC

**Figure 4:**
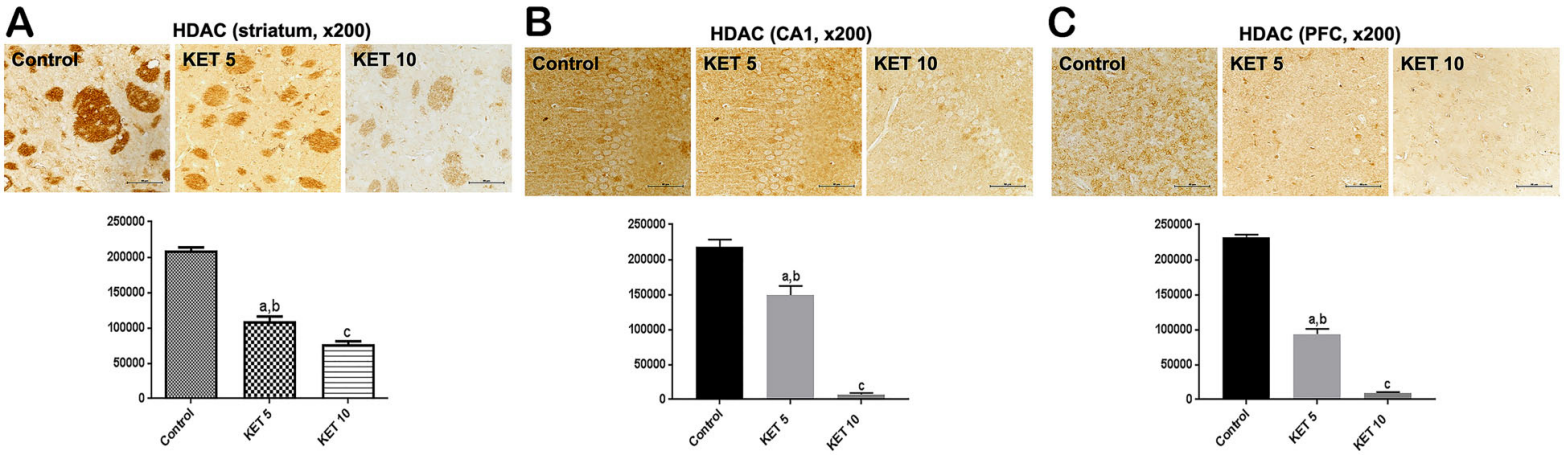
Single administrations of ketamine (KET, 5 and 10 mg/kg, *ip*) significantly reduced histone deacetylase (HDAC) immunoreactivities in the **A**) striatum, **B**) CA1 hippocampal subfield, and **C**) prefrontal cortex (PFC) compared with controls (3 animals per group). Representative photomicrographs, at ×200 magnification (50 μm), taken at D1 protocol. The graphs show the relative absorbance determined by the ImageJ software (NIH, USA). Data are reported as means±SE. ^a,b^P<0.01 *vs* Control; ^c^P<0.01 *vs* KET 5 (one-way ANOVA and Tukey as the *post hoc* test).

For HDAC, 45 and 61% decreases were observed in the striata after single KET administration at the doses of 5 and 10 mg/kg, respectively, compared with the controls [F(2,9)=155.9, P<0.0001] (Figure 4A). The CA1 hippocampal subfield presented 32 and 97% decreases, compared with the control group [F(2,12)=119.6, P<0.0001] (Figure 4B). The PFC showed 60 and 96% decreases, respectively, at the KET doses of 5 and 10 mg/kg [F(2,12)=563.1, P<0.0001] (Figure 4C).

### Immunohistochemical results for BDNF

**Figure 5:**
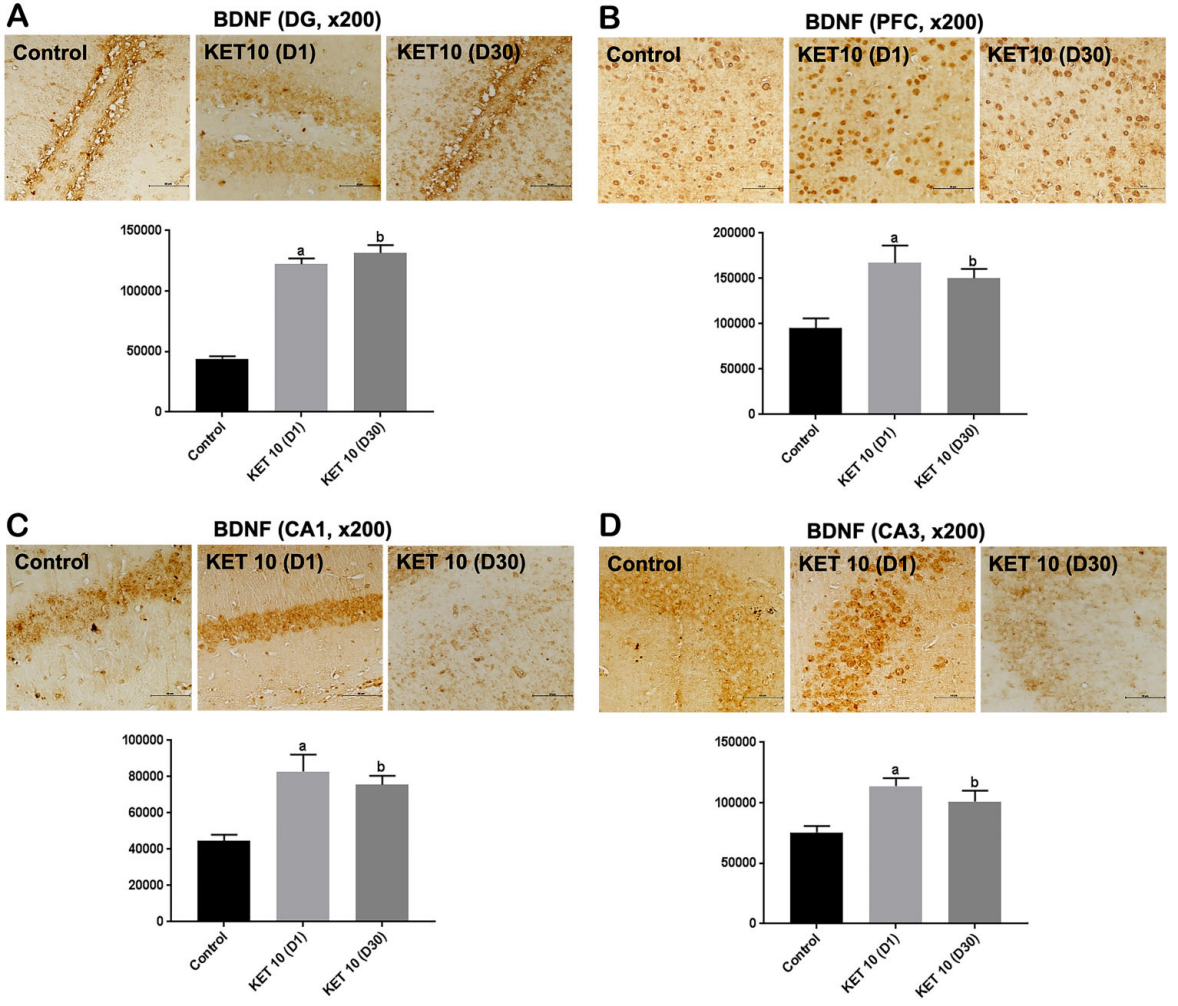
Single administrations of ketamine (KET, 10 mg/kg, *ip*) significantly increased brain-derived neurotrophic factor (BDNF) expression in the **A**) dentate gyrus (DG), **B**) prefrontal cortex (PFC), **C**) CA1, and **D**) CA3 hippocampal subfields compared with controls (3 animals per group). Representative photomicrographs, at ×200 magnification (50 μm), taken at D1 and D30 protocols. The graphs show the relative absorbance determined by the ImageJ software (NIH, USA). Data are reported as means±SE. DG: ^a,b^P<0.05 *vs* Control; PFC: ^a,b^P<0.01 *vs* Control; CA1: ^a,b^P<0.05 *vs* Control; CA3: ^a,b^P<0.05 *vs* Control (one-way ANOVA and Tukey as the *post hoc* test).

The neurotrophin BDNF is considered a link between the antidepressant drug and the neuroplastic changes, resulting in the improvement of depression (11). This led us to verify whether the increase of this neurotrophin in the brain could, at least partly, explain the long-lasting effects of KET. BDNF immunoreactivities increased almost 3-times in the DG at both time-points in relation to controls [F(2,15)=7282, P<0.0001] (Figure 5A). In addition, increases of around 2-times were observed in the CA1 subfield, at D1 and D30, respectively, compared with the control group [F(2,15)=12.69, P<0.0006] (Figure 5B). Similar increases (1.6- and 1.4-times, respectively) were demonstrated in the CA3 area, for both time-points [F(2,14)=7.895, P<0.0051] (Figure 5C). More importantly, 2- and 4-times increases, in a time-dependent manner, were observed in the PFC, at D1 and D30, respectively, compared with the controls [F(2,12)=63.17, P<0.0001] (Figure 5D). These results suggested that KET-induced increases in brain BDNF are well maintained even after 30 days of a single KET injection and may explain the long-lasting antidepressant effect of the drug.

### Immunohistochemical results for brain GFAP

**Figure 6:**
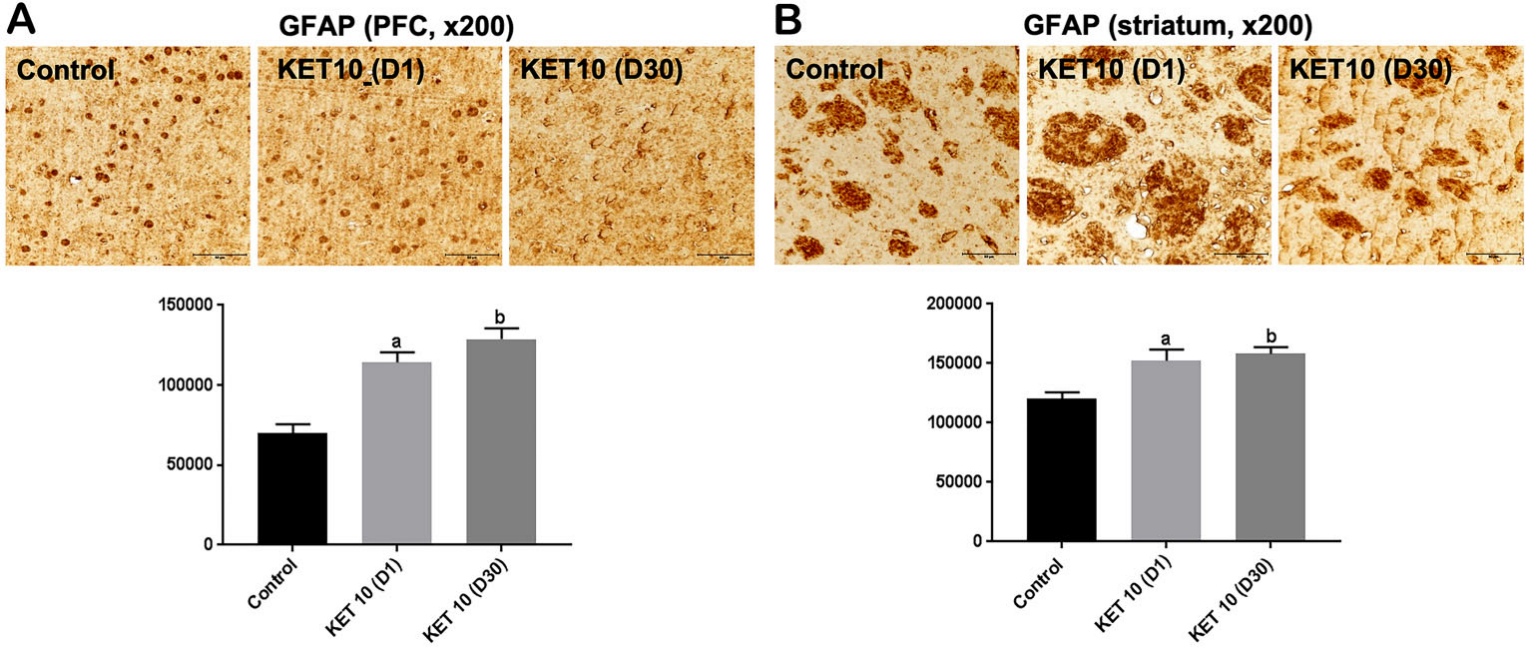
Single administrations of ketamine (KET, 10 mg/kg, *ip*) significantly increased brain glial fibrillary acidic protein (GFAP) expression in the **A**) prefrontal cortex (PFC) and **B)** striatum compared with controls (3 animals per group). Representative photomicrographs at ×200 magnification (50 μm), taken at D1 and D30 protocols. The graphs show the relative absorbance determined by the ImageJ software (NIH, USA). Data are reported as means±SE. ^a,b^P<0.05 *vs* Control (one-way ANOVA and Tukey as the *post hoc* test).

Astrocytes are the most abundant cells in the brain, participating in most brain functions. Evidence from preclinical studies has revealed morphological and functional astrocyte alterations in animal models of depression, which has been confirmed in *postmortem* studies (13). KET 10 (D1) significantly increased GFAP staining in the PFC by 1.6-times, compared with the control group. A similar increase (1.8-times) was observed at D30 [F(2,12)=24.79, P<0.0001] (Figure 6A). Interestingly, higher effects were observed at D30 in another cortical area (CTX) and a dose-effect relationship was seen after the injections of KET (5 and 10 mg/kg, at D30), compared with the control group (data not shown). In addition, significant increases (around 1.3-times) were also observed in the striatum at D1 and D30 (Figure 6B).

## Discussion

We previously showed a dose-dependent antidepressant-like effect of KET in mice, at subanesthetic doses (5 and 10 mg/kg, *ip*). In addition, the effect of a lower dose (2 mg/kg) was significantly increased by KET combination with lithium, evaluated by the FST (15). This synergistic effect was probably the result of inhibitions of GSK-3 and HDAC, important targets for both drugs. Recently, we also demonstrated that the fast antidepressant effects of KET are, at least partly, due to its action on striatal monoaminergic pathways (7).

In the present study, we showed that single injections of subanesthetic doses of KET (5 and 10 mg/kg) significantly decreased the immobility time, evaluated by the FST, even 30 days later, suggesting a sustained antidepressant-like effect. Similar effects, also evaluated by the FST, were observed by others who showed the antidepressant-like effects of a single injection of KET (10 or 30 mg/kg), 1 and 7 days later (17).

Here, we focused mainly on KET long-lasting antidepressant-like effects and on GSK-3, HDAC, BDNF, and GFAP expressions in the brain, after injections of single low doses. GSK-3 is an important serine-threonine kinase, abundant in the central nervous system. Its activity is inhibited through phosphorylation of serine 21 in GSK-3 alpha and serine 9 in GSK-3 beta (18). This enzyme is involved in cellular functions, such as metabolism, transcription, cell survival, and synaptic plasticity. The dysregulation of GSK-3 could have multiple effects, leading to impaired neural plasticity and gene expression, and the ability of neurons to respond to stressful conditions. Most importantly, inhibition of GSK-3 was demonstrated to be required for the rapid antidepressant effect of KET in mice, augmenting the signaling through AMPA receptors (19).

Furthermore, GSK-3 beta seems to selectively regulate depression, memory, and hippocampal cell proliferation (20). In the present work, we demonstrated very high GSK-3 inhibition (up to 99%) in all brain areas tested (striatum, dentate gyrus, CA1, and, especially, PFC), after single injections of KET in male mice. GSK-3 has been shown to be a key regulator for KET rapid antidepressant-like effects (21). However, the mechanisms of KET action go beyond the NMDA blockade, may act in a complementary way, and are not necessarily exclusive (22).

The modulation of gene expression is an important process for the mammalian brain function and, thus, a way for regulating gene expression is through chromatin remodeling. The acetylation of histones by acetyl transferases (HATs) and HDACs are the most studied histone post-translational modifications in cognition and neuropsychiatric diseases (23). Evidence indicates that brain HDAC inhibition may provide an epigenetic-based target for improved treatments of mood disorders (24).

Furthermore, MDD results from gene and environmental interactions, mediated by epigenetic mechanisms, such as chromatin and DNA modifications altering gene expression (25). Importantly, these epigenetic mechanisms may be involved in the role of BDNF in depression and response to antidepressants. Preclinical studies demonstrated the antidepressant-like efficacy of HDAC inhibitors. For instance, the systemic administration of a HDAC inhibitor rescues, in part, the depressive-like behavior of the CREB-regulated transcription co-activator 1-deficient mice (26).

The stress-reduced hippocampal BDNF expression has been already demonstrated, and this reduction is prevented by antidepressants. Similar changes occur in the brain of patients with MDD (27). BDNF is a key player in the antidepressant action and considered a transducer, acting as a link between the antidepressant and neuroplastic changes that result in the improvement of depressive symptoms (28). In the present study, we showed that increases in BDNF expression persisted up to 30 days after KET administration in DG, CA1, and CA3 subfields. However, and most importantly, these antidepressant-like changes occurred mainly at the PFC and in a time-dependent manner, at the 60 min (D1) and 30-day (D30) time-points, after the single KET administration.

BDNF actions are regulated by neuronal activity and these can lead to trophic effects, as formation, stabilization, and potentiation of synapses, through the high affinity of BDNF for the tropomyosin receptor kinase B (Trk-B) receptors (29). Recent data (30) suggest that the prolonged antidepressant effects, observed after a single KET infusion to depressed patients refractory to treatment, can be related to a transient enhancement of neuroplasticity, induced by a glutamate burst in some brain neurons, which may, at least in part, explain the results of the present study.

Astrocytes coordinate synaptic networks and are the most abundant cell type in the brain, participating in the majority of brain functions. Evidence supports the observation that depression is associated with a decreased density and hypofunction of astrocytes observed in MDD patients and in animal models of depression (31). Thus, these events are expected to contribute to synaptic dysfunction present in depressive-like conditions. Astrocytes are important in the information processing in the brain, modulating synaptic activity and plasticity, and their dysfunction may contribute to smaller hippocampal volume in MDD (32).

In the present work, we showed that KET increased GFAP immunoreactivity in the PFC and these changes were observed at D1 and D30, after a single KET administration. A greater and time-dependent increase in GFAP expression was also demonstrated in another brain cortical area. The loss of glia cells in prefrontal and limbic brain regions was observed in depressed patients and in animal models, and the degeneration of astrocytes is known to result in glutamate excess in the synaptic cleft and glutamate/GABA imbalance in affected structures (33). Furthermore, evidence from postmortem human brain studies highlight changes in glial cell morphology, astrocyte-related biomarkers, and genes, following mood disorders, thus suggesting astrocytes as a promising target for mood disorder interventions (34).

Moreover, neural plasticity plays a significant role in the onset and development of depression, and evidence indicates that the PFC is an extremely plastic brain area (35). Lesions of the PFC in rats were shown to result in depressant-like behavior and, in addition, the PFC is considered a target for antidepressant drugs, including KET. Furthermore, a single sub-anesthetic dose of KET leads to fast antidepressant effects and, in rodent models, is associated with higher dendritic spine density in the prefrontal cortex (36).

Although the question on sustained KET effects is still a matter of controversy, evidence has shown the long-lasting effects of a subcutaneous dose of KET (0.2 mg/kg) in a melancholic depression patient who remained in remission for 5 months (37). Our study showed, for the first time, not only the rapid but also the long-lasting KET effects (up to 30 days), after the administration of single doses to male mice. KET rapid effects are associated to brain GSK-3 and HDAC inhibitions. However, not only the rapid effects but also the long-lasting antidepressant effects of KET show a close relationship to the increases in brain BDNF levels and GFAP expression. These increases were observed in the hippocampus and PFC.

Differences between short- and long-term responses to KET were studied in a genetic model of depression in rats (38). The authors showed that KET rapid response entailed robust and strain-independent topological modifications in cognitive, sensory, emotion, and reward-related circuitry, including regions that exhibited correlation of connectivity metrics with depressive behavior. On the other hand, the strain-specific long-term effects of KET included normalization of connectivity measures for habenula and midline thalamus. In this cognitive model of depression, the authors suggest that KET mediates its pro-cognitive effects by normalizing the disrupted wiring within the habenula-mid-thalamic-hippocampal cognitive circuitry, which might be a key imaging correlate of KET long-term effects.

The identification of KET mechanisms of action, responsible for its long-lasting effects, will certainly impact the pharmacological strategies for treating major depressive disorders in the future. Neuronal plasticity seems to be a central mechanism in the action of antidepressant drugs and may be responsible for KET long-lasting effects. For this, KET ability to modulate neuroplasticity through its action on BDNF expression could certainly be a critical event. However, a limitation of the present study is related to the use of naive mice and an acute stress model as the FST, instead of models focusing on chronic mild stress or chronic unpredictable stress.

In conclusion, antidepressant drugs rapidly activate TrkB signaling and increase BDNF expression and neuronal plasticity, indicating that this could be a mechanism responsible for the KET long-lasting antidepressant action. In addition, evidence shows that astroglial atrophy contributes to the pathophysiology of depression, thus a morphological modification of astrocytes could also respond to KET antidepressant actions (39). Indeed, antidepressants have been demonstrated to reactivate a state of plasticity in the adult cortex, resembling the enhanced plasticity observed during postnatal periods (40), which could certainly explain, at least partly, the results of the present study.
